# Estrogen Exposure, Metabolism, and Enzyme Variants in a Model for Breast Cancer Risk Prediction

**DOI:** 10.4137/cin.s2262

**Published:** 2009-05-05

**Authors:** Fritz F. Parl, Kathleen M. Egan, Chun Li, Philip S. Crooke

**Affiliations:** 1 Department of Pathology, Vanderbilt University, Nashville, TN 37232; 3 Department of Biostatistics, Vanderbilt University, Nashville, TN 37232; 4 Department of Mathematics and Integrative Cancer Biology Center, Vanderbilt University, Nashville, TN 37232; 2 Division of Cancer Prevention and Control, H. Lee Moffitt Cancer Center and Research Institute, Tampa FL 33612

**Keywords:** breast cancer, risk assessment, hormonal carcinogenesis, computational biology, model

## Abstract

Estrogen is a well-known risk factor for breast cancer. Current models of breast cancer risk prediction are based on cumulative estrogen exposure but do not directly reflect mammary estrogen metabolism or address genetic variability between women in exposure to carcinogenic estrogen metabolites. We are proposing a mathematical model that forecasts breast cancer risk for a woman based on three factors: (1) estimated estrogen exposure, (2) kinetic analysis of the oxidative estrogen metabolism pathway in the breast, and (3) enzyme genotypes responsible for inherited differences in the production of carcinogenic metabolites. The model incorporates the main components of mammary estrogen metabolism, i.e. the conversion of 17β-estradiol (*E*_2_) by the phase I and II enzymes cytochrome P450 (*CYP*) *1A1* and *1B1*, catechol-O-methyltransferase (*COMT*), and glutathione S-transferase P1 (*GSTP1*) into reactive metabolites, including catechol estrogens and estrogen quinones, such as *E*_2_-3,4-*Q* which can damage DNA. Each of the four genes is genotyped and the SNP data used to derive the haplotype configuration for each subject. The model then utilizes the kinetic and genotypic data to calculate the amount of *E*_2_-3,4-*Q* carcinogen as ultimate risk factor for each woman. The proposed model extends existing models by combining the traditional “phenotypic” measures of estrogen exposure with genotypic data associated with the metabolic fate of *E*_2_ as determined by critical phase I and II enzymes. Instead of providing a general risk estimate our model would predict the risk for each individual woman based on her age, reproductive experiences as well as her genotypic profile.

## Introduction

Estrogens have long been recognized as the primary risk factor for the development of breast cancer.^1^,^2^ Epidemiologic studies have indicated that breast cancer risk is higher in women with early menarche and late menopause, who have longer exposure to estrogens.^3^ A pooled analysis of nine prospective studies found that circulating estrogen levels were directly related to risk of breast cancer in postmenopausal women.^4^ Based on these data, current models of breast cancer risk prediction are mainly based on cumulative estrogen exposure and include such factors as age, age at menarche, and age at first live birth;^5^,^6^ (www.cancer.gov/bcrisktool). While all these studies implicate estrogens as risk factor for the development of breast cancer, they leave open two important questions that need to be answered to advance from an empirical, global risk assessment to a truly etiological, individualized assessment. The questions are: (1) How do estrogens cause breast cancer? and (2) Since all women are exposed to estrogens, how do we better delineate risk? To close these gaps in our knowledge we need to explain mechanisms of estrogen carcinogenesis and inter-individual risk variation and our approach is to examine the dynamics of a pathway for estrogen metabolism and use its prediction of the level of DNA corrupting compounds as a predictor of breast cancer risk.

Carcinogenesis is usually viewed as a stepwise process beginning with genotoxic effects (initiation) followed by enhanced cell proliferation (promotion). The main estrogen, 17β-estradiol (*E*_2_ ), is a substrate for the phase I enzymes, cytochrome P450 (*CYP*) *1A1* and *1B1* and a ligand for the estrogen receptor. In its dual role of substrate and ligand, *E*_2_ has been implicated in the development of breast cancer by simultaneously causing DNA damage via its oxidation products, the *2-OH* and *4-OH* catechol estrogens, and by stimulating cell proliferation and gene expression via the estrogen receptor. Thus, *E*_2_ and its oxidative metabolites are unique carcinogens that affect both tumor initiation and promotion.^7^–^9^

**Figure 1:**
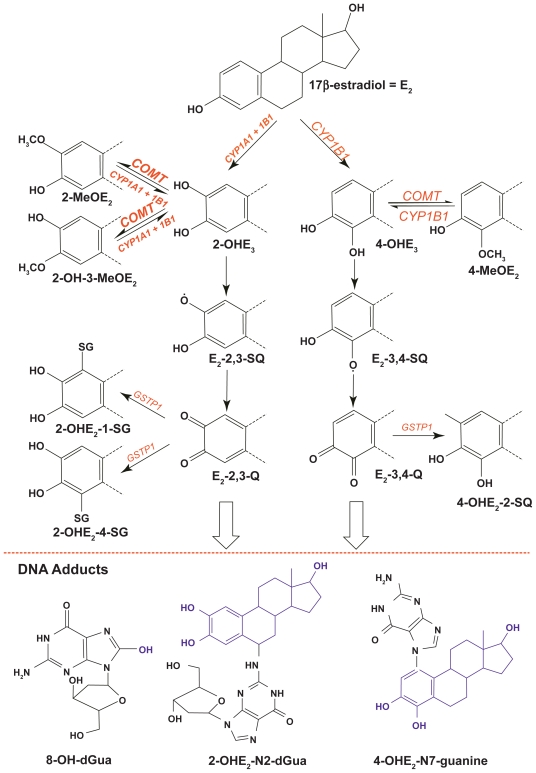
*Oxidative estrogen metabolism causes DNA adduct formation*. The estrogen metabolism pathway is regulated by oxidizing phase I and conjugating phase II enzymes. *CYP1A1* and *CYP1B1* catalyze the oxidation of *E*_2_ to catechol estrogens 2-OH*E*_2_ and 4-OH*E*_2_. The catechol estrogens are either methylated by *COMT* to methoxyestrogens (2*-*MeO*E*_2_, 2-OH-3*-*MeO*E*_2_, 4*-*MeO*E*_2_) or further oxidized by *CYP*s to semiquinones (*E*_2_-2,3-SQ, *E*_2_-3,4-SQ) and quinones (*E_2_-2,3-Q, E_2_-3,4-Q*). The estrogen quinones are conjugated by *GSTP1* to GSH-conjugates (2-OH*E*_2_-1-SG, 2-OH*E*_2_-4-SG, 4-OH*E*_2_-2-SG). Alternatively, the quinones can form quinone-DNA adducts (e.g. 4-OH*E*_2_-N7-guanine, 2-OH*E*_2_-N2-deoxyguanosine) or cause oxidative adducts (e.g. 8-OH-deoxyguanosine) via reactive oxygen species resulting from redox-cycling between semiquinones and quinones. The three adducts and their estrone (*E*_1_) and adenine counterparts have been identified in human breast tissues.^56^,^57^ Recently, we demonstrated experimentally that *CYP1B1*-mediated oxidation of *E*_2_ in the presence of deoxyguano-sine caused the formation of the 4-OHE_2_-N7-guanine adduct.^48^ Our results provide direct evidence that metabolism of the parent hormone can initiate DNA damage.

As shown in Figure 1, *E*_2_ is oxidized to catechol estrogens by *CYP1A1* and *CYP1B1*. These enzymes further oxidize the catechol estrogens to semiquinones and quinones. The highly reactive estrogen quinones form Michael addition products with deoxyribonucleosides.^10^–^12^ Thus, estrogen quinones share a common feature of many chemical carcinogens, i.e. the ability to covalently modify DNA.^13^–^16^ Furthermore, estrogen semiquinones and quinones undergo redox-cycling, which results in the production of reactive oxygen species that can cause oxidative DNA damage.^17^–^19^

Support for the carcinogenic activity of estrogens and their oxidative products, the catechol estrogens, comes from experiments in animal models. Treatment with either *E*_2_ or the *2-OH* or *4-OH* catechol estrogens caused kidney cancer in male Syrian hamsters and endometrial cancer in female CD1 mice, the latter compounds being the most carcinogenic agents.^20^–^22^ However, there is no animal model for estrogen-induced breast cancer and even in the hamster and mouse models the precise mechanism of DNA damage is uncertain. Thus, there is a need to understand estrogen metabolism in the human breast in order to elucidate the role of endogenous and exogenous estrogens in mammary carcinogenesis. To advance this understanding requires not only characterization of the various estrogen metabolites but equally important, a precise definition of the responsible enzymes. Several investigators have proposed a qualitative model of mammary estrogen metabolism regulated by oxidizing phase I and conjugating phase II enzymes.^23^,^24^ The oxidative estrogen metabolism pathway starts with *E*_2_ and *E*_1_, which are oxidized to the *2-OH* and *4-OH* catechol estrogens by the phase I enzymes *CYP1A1* and *CYP1B1*.^25^,^26^ As described above, the P450-mediated estrogen metabolism is expected to lead to the formation of both estrogen and oxidative DNA adducts, all of which have been shown to possess mutagenic potential.^27^,^28^ It is postulated that the genotoxicity of the oxidative estrogen metabolism pathway is mitigated by alternate reactions of the metabolites with phase II enzymes. Specifically, catechol-O-methyl transferase (*COMT*) catalyzes the methylation of catechol estrogens to methoxy estrogens, which lowers the catechol estrogens available for conversion to estrogen quinones.^29^,^30^ In turn, the estrogen quinones undergo conjugation with glutathione (GSH) via the catalytic action of glutathione S-transferase *GSTP1*.^31^,^32^ The formation of GSH-estrogen conjugates would reduce the level of estrogen quinones and thereby lower the potential for DNA damage.

The current models of mammary estrogen metabolism have limitations. Firstly, only single enzymes, e.g. *CYP1B1* and *COMT*, have been analyzed to date with simple substrate-product kinetics, which clearly generates an incomplete picture of the metabolic pathway. Secondly, while the model incorporates the functional roles of the phase I and II enzymes, it does so only qualitatively and it remains uncertain how the enzymes interact quantitatively. Third, each of the phase I and II enzymes contains genetic polymorphisms.^26^,^29^,^33^,^34^ Studies from several laboratories have examined the functional implications of the polymorphisms on estrogen metabolism, again focusing on single enzymes.^26^,^29^,^30^,^35^,^36^ Thus, the multitude of potential kinetic reactions resulting from the complex genetic variations of the phase I and II enzymes is completely outside the scope of the current model of estrogen metabolism. In contrast to the relatively small number of functional studies of estrogen metabolism, multiple epidemiological studies have investigated breast cancer risk in relation to genetic variation in the critical enzymes involved in estrogen metabolism with inconsistent findings.^37^,^38^ A drawback of any purely genetic assessment is the lack of information about functional interactions inherent in complex metabolic pathways such as the estrogen metabolism pathway. Thus, such studies cannot assess the underlying metabolic interactions in the pathway.^39^,^40^ A pathway-based functional and quantitative approach is necessary to overcome the current limitation in genotype assessment.

We have developed an experimental *in vitro* model of mammary estrogen metabolism, in which we combined purified, recombinant phase I enzymes *CYP1A1* and *CYP1B1* with the phase II enzymes *COMT* and *GSTP1* to determine how *E*_2_ is metabolized.^41^ We employed both gas and liquid chromatography with mass spectrometry (GC/MS and LC/MS) to measure the parent hormone *E*_2_ as well as eight metabolites, i.e. the catechol estrogens, methoxyestrogens, and estrogen-GSH conjugates. With this important experimental data, an *in silico* model of the metabolic pathway has been developed.^42^

## Methods

A mathematical model for the estrogen metabolism pathway that is shown in Figure 1 can be constructed using some basic assumptions about the kinetics of the reactions in this figure. We assume that each reaction in the pathway (*A* → *B*, a generic step in the pathway) is an enzyme-catalyzed reaction of the form: $A+E\overset{k_{1}}{\underset{k_{2}}{⇄}}C\overset{k_{3}}{\to }B+E$ where *E* denotes the enzyme, *C* is the enzyme-substrate complex, and *k_i_*_,_ *i =* 1,2,3, are the rate constants of the reaction. For these types of reaction we approximate the kinetics using the quasi-steady state assumption: *C* = *E* *A /(*K_m_* *+ A*), *K_m_* *=* (*k*_2_ *+ k*_3_) /*k*_1_ , where E* is the initial enzyme concentration. We can “prove” this approximation by looking at the differential equations for $A+E\overset{k_{1}}{\underset{k_{2}}{⇄}}C\overset{k_{3}}{\to }B+E$:

$$\begin{array}{l} \frac{dA}{dt}=-k_{1}AE+k_{2}C,A(0)=A_{0}\\ \frac{dB}{dt}=k_{3}C,B(0)=0\\ \frac{dC}{dt}=k_{1}AE-(k_{2}+k_{3})C,C(0)=0\\ \frac{dE}{dt}=-k_{1}AE+(k_{2}+k_{3})C,E(0)=E_{0} \end{array}$$

This system of differential equations yields two conservation laws:

$$\begin{array}{l} A(t)+C(t)+B(t)=A_{0}\\ C(t)+E(t)=E_{0} \end{array}$$

With the conservation laws, we can reduce the four differential equations to two differential equations:

$$\begin{array}{l} \frac{dA}{dt}=-k_{1}E_{0}S+(k_{1}A+k_{2})C,A(0)=A_{0}\\ \frac{dC}{dt}=k_{1}E_{0}A-(k_{1}A+k_{2}+k_{3})C,C(0)=0\\ \Rightarrow \frac{dC}{dA}=\frac{k_{1}E_{0}A-(k_{1}A+k_{2}+k_{3})C}{-k_{1}E_{0}S+(k_{1}A+k_{2})C}. \end{array}$$

We now assume that the reaction has progressed to the state that

$$\begin{array}{l} \frac{dC}{dA}\approx 0\Rightarrow k_{1}E_{0}A=(k_{1}A+k_{2}+k_{3})C\\ \Rightarrow C(A)=\frac{k_{1}E_{0}A}{k_{1}A+k_{2}+k_{3}}=\frac{E_{0}A}{\frac{k_{2}+k_{3}}{k_{1}}+A}\\ =\frac{E_{0}A}{K_{m}+A},\;K_{m}=\frac{k_{2}+k_{3}}{k_{1}}. \end{array}$$

Hence, the formation of *B*(*t*) is approximately given by *dB/dt* ≈ *k*_3_ (*E*_0_*A/K_m_* *+ A*) = *k_cat_* E*A/(*K_m_* *+ A*) where *k_cat_* *= k*_3_ and E* = *E*_0_ is the initial enzyme level.

More information about the quasi-steady state approximation can be found in Parl et al.^43^ Using this approach for the individual reactions in Figure 1, we can write down to a system of nonlinear, ordinary differential equations for the concentrations of the compounds in the pathway:

(1) $$\begin{array}{l} \frac{d(E_{2})}{dt}=-\frac{k_{cat_{1}}E_{CYP1B1}E_{2}}{K_{m_{1}}+E_{2}}-\frac{k_{cat_{2}}E_{CYP1A1}E_{2}}{K_{m_{2}}+E_{2}}\\ -\frac{k_{cat_{3}}E_{CYP1B1}E_{2}}{k_{m_{3}}+E_{2}} \end{array}$$

(2) $$\begin{array}{l} \frac{d({OHE}_{2}^{2})}{dt}=\frac{k_{cat_{2}}E_{CYP1A1}E_{2}}{k_{m_{2}}+E_{2}}+\frac{k_{cat_{3}}E_{CYP1B1}E_{2}}{k_{m_{3}}+E_{2}}-\frac{k_{cat_{6}}E_{COMT}{OHE}_{2}^{2}}{K_{m_{6}}+{OHE}_{2}^{2}}+\frac{k_{cat_{7}}E_{CYP1A1}{MeOHE}_{2}^{2}}{k_{m_{7}}+{MeOHE}_{2}^{2}}\\ +\frac{k_{cat_{8}}E_{CYP1B1}{MeOHE}_{2}^{2}}{k_{m_{8}}+{MeOHE}_{2}^{2}}-\frac{k_{cat_{9}}E_{COMT}{OHE}_{2}^{2}}{k_{m_{9}}+{OHE}_{2}^{2}}+\frac{k_{cat_{10}}E_{CYP1A1}{MeOHE}_{2}^{23}}{k_{m_{10}}+{MeOHE}_{2}^{23}}\\ +\frac{k_{cat_{11}}E_{CYP1B1}{MeOHE}_{2}^{23}}{K_{m_{11}}+{MeOHE}_{2}^{23}}-\frac{V_{{\text{max}}_{Q1}}{\left({OHE}_{2}^{2}\right)}^{\alpha }}{K_{m_{Q1}}+{\left({OHE}_{2}^{2}\right)}^{\alpha }} \end{array}$$

(3) $$\frac{d({OHE}_{2}^{4})}{dt}=\frac{k_{cat_{1}}E_{CYP1B1}E_{2}}{K_{m_{1}}+E_{2}}-\frac{k_{cat_{4}}E_{COMT}{OHE}_{2}^{4}}{K_{m_{4}}+{OHE}_{2}^{4}}+\frac{k_{cat_{5}}E_{COMT}{MeOHE}_{2}^{4}}{K_{m_{5}}+{MeOHE}_{2}^{4}}-\frac{V_{{\text{max}}_{Q2}}{\left({OHE}_{2}^{4}\right)}^{\beta }}{K_{m_{Q2}}+{\left({OHE}_{2}^{4}\right)}^{\beta }}$$

(4) $$\frac{d({MeOHE}_{2}^{4})}{dt}=\frac{k_{cat_{4}}E_{COMT}{OHE}_{2}^{4}}{K_{m_{4}}+{OHE}_{2}^{4}}-\frac{k_{cat_{5}}E_{CYP1B1}{MeOHE}_{2}^{4}}{K_{m_{5}}+{MeOHE}_{2}^{4}}$$

(5) $$\frac{d({MeOHE}_{2}^{2})}{dt}=\frac{k_{cat_{6}}E_{COMT}{OHE}_{2}^{2}}{K_{m_{6}}+{OHE}_{2}^{2}}-\frac{k_{cat_{7}}E_{CYP1A1}{MeOHE}_{2}^{2}}{K_{m_{7}}+{MeOHE}_{2}^{2}}-\frac{k_{cat_{8}}E_{CYP1B1}{MeOHE}_{2}^{2}}{k_{m_{8}}+{MeOHE}_{2}^{2}}$$

(6) $$\frac{d({MeOHE}_{2}^{23})}{dt}=\frac{k_{cat_{9}}E_{COMT}{OHE}_{2}^{2}}{K_{m_{9}}+{OHE}_{2}^{2}}-\frac{k_{cat_{10}}E_{CYP1A1}{MeOHE}_{2}^{23}}{K_{m_{10}}+{MeOHE}_{2}^{23}}-\frac{k_{cat_{11}}E_{CYP1B1}{MeOHE}_{2}^{23}}{K_{m_{11}}+{MeOHE}_{2}^{23}}$$

(7) $$\frac{d({EQ}_{2}^{23})}{dt}=\frac{V_{{\text{max}}_{Q1}}{\left({OHE}_{2}^{2}\right)}^{\alpha }}{K_{m_{Q1}}+{\left({OHE}_{2}^{2}\right)}^{\alpha }}-\frac{k_{cat_{13}}E_{GSTP1}{EQ}_{2}^{23}}{K_{m_{13}}+{EQ}_{2}^{23}}-\frac{k_{cat_{14}}E_{GSTP1}{EQ}_{2}^{23}}{K_{m_{14}}+{EQ}_{2}^{23}}-k_{1}{EQ}_{2}^{23}$$

(8) $$\frac{d({EQ}_{2}^{34})}{dt}=\frac{V_{{\text{max}}_{Q2}}{\left({OHE}_{2}^{4}\right)}^{\beta }}{K_{m_{Q2}}+{\left({OHE}_{2}^{4}\right)}^{\beta }}-\frac{k_{cat_{12}}E_{GSTP1}{EQ}_{2}^{34}}{K_{m_{12}}+{EQ}_{2}^{34}}-k_{2}{EQ}_{2}^{34}$$

(9) $$\frac{d({OHE}_{2}^{21}SG)}{dt}=\frac{k_{cat_{14}}E_{GSTP1}{EQ}_{2}^{23}}{K_{m_{14}}+{EQ}_{2}^{23}}$$

(10) $$\frac{d({OHE}_{2}^{24}SG)}{dt}=\frac{k_{cat_{13}}E_{GSTP1}{EQ}_{2}^{23}}{K_{m_{13}}+{EQ}_{2}^{23}}$$

(11) $$\frac{d({OHE}_{2}^{42}SG)}{dt}=\frac{k_{cat_{12}}E_{GSTP1}{EQ}_{2}^{34}}{K_{m_{12}}+{EQ}_{2}^{34}}$$

Here *k_cat_*_*_j_*_ and *k_m_*_*_j_*_ are constants and *E_enzyme_* are the enzyme levels in the reactions. There are parts of the pathway for which kinetic data is not available. In particular, rate constants are not known for the reactions: 2-*OHE*_2_ → *E*_2_-2,3-*SQ* → *E*_2_-2,3-*Q* and 4-*OHE*_2_ → *E*_2_-3,4-*SQ* → *E*_2_-3,4-*Q* reactions. Our first simplification is to collapse these reaction to single reactions, 2-*OHE*_2_ → *E*_2_-2,3-*Q* and 4-*OHE*_2_ → *E*_2_-3,4-*Q*, respectively. The next simplification is to assume that each of these quinone production reactions (*OHE*_2_*^k^* → *EQ*_2_*^ij^*) satisfy dynamics of the form: *dEQ*_2_*^ij^/dt* = *V*_max*_Q_*_ (*OHE*_2_*^k^*)*^σ^* / *K_m_*_*_Q_*_ + (*OHE*_2_*^k^*) *^σ^* where *V*_max*_Q_*,_ *k_m_*_*_Q_*_ and σ are constants. For the mathematical model to be a tractable computational model of the metabolism pathway, it is necessary to have estimates of these unknown constants. We next look at a technique for estimating *α*, *β*, *V*_max*_Q_*_1__, *V*_max*_Q_*_2__, *K_mQ_*_1_*, and K_m_*_*_Q_*_2_._

Experimental values for rate constants of the *CYP1A1*, *CYP1B1*, *COMT*, and *GSTP1* catalyzed reactions are available.^26^,^29^,^32^,^41^,^42^ Furthermore, the concentrations over time for each non-quinone compound in the complete pathway have been measured for a particular starting concentration of *E*_2_ i.e. *E*_2_(0) = *E*_2_0__.^41^ Using this data, a searching algorithm was written in *Mathematica* (Wolfram Research, Inc.) to find values for *V*_max*Q*_*K*_*mQ*_ and *σ* in each of the two quinones reactions that fit the experimental data in a certain metric using numerical solutions of the differential equation system. The constants, *α*, *β*, *V*_max_*Q*1__, *V*_max_*Q*2__, *K*_*mQ*1_ and *K*_*mQ*2_, were obtained in this manner.

## Results

**Figure 2:**
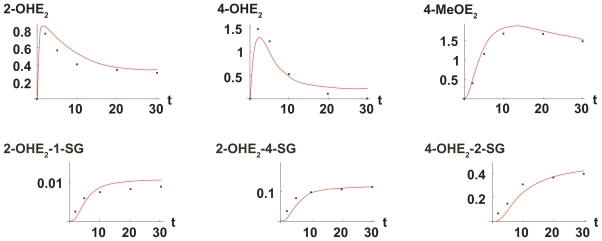
*Comparison of mathematical model with experimental data.* The red curves are plots of the solutions to the nonlinear system of differential equations and the blue dots are experimental data.^41^ As shown, the model allowed simulations of all reactions in the pathway, which agreed well with the experimentally determined results.^42^

Figure 2 shows comparisons between the model (solid curves) and the data^41^ over a simulation covering 30 minutes for some of the components in the pathway. These simulations used the estimates for *α*, *β*, *V*_max*_Q_*_1__, *V*_max*_Q_*_2__, *K_mQ_*_1_, and *K_m_*_*_Q_*_2__ calculated above as well as the published values of the other kinetic parameters. In the simulations of the pathway it was assumed that initially all quantities are zero, except for *E*_2_ (0) = *E*_2_0__. Enzyme concentrations used in the simulation are consistent with those reported previously.^41^

**Figure 3:**
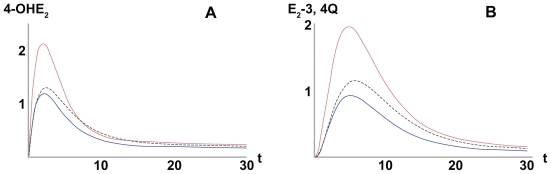
*Kinetic-genomic modeling of catechol estrogen.* (**A**) 4*-OHE*_2_ and estrogen quinone (**B**) *E*_2_*-*3,4*-Q* using rate constants for wild-type and variant *CYP1A1*, *CYP1B1*, and *COMT*. The Area Under the Curve = *AUC* represents the metabolite production over time. Only the highest, lowest, and wild-type (dotted line) *AUC*s are shown.^42^

Having all of the parameters of the system, one can view the model as giving functional relations between *E*_2_(*t*) and the estrogen quinone concentrations: *EQ*_2_^23^ (*t*) and *EQ*_2_^34^ (*t*). Figure 3 shows the time-wise buildup and decay of the estrogen quinones. In an attempt to give a simple measure of the quinone concentrations over the course of time, we introduce the *Area* *Under the* *Curve* (*AUC*) metric: *AUC_k_* = ∫*_0_^T^* *EQ*_2_*^k^* (*t*) *d* where *k* = 23,24 and *T* = 30 min. It is possible to introduce other measures e.g. ${EQ}_{2\text{max}}^{ij}=\underset{0\leq t\leq T}{\text{max}}{EQ}_{2}^{ij}(t)$ which is the highest concentration achieved during the time interval [0, *T*]. We have chosen the *AUC* metric because it incorporates both concentration level and time.

The mathematical model for the estrogen metabolism pathway provides a relationship between an input *E*_2_ and two outputs *AUC*_23_ and *AUC*_24_. It can also be view as connecting the area under the curve outcomes to the kinetic parameters, *k_cat_* and *K_m_*, embedded in the model. The model permits one to analyze the behavior of the area under curve variables as functions of the kinetic parameters, either for a single step in the pathway or a combination of steps. This analysis allows one to view how variations in the kinetic parameters, which are the result of polymorphism of the enzymes, affect the area under the curve outcomes.

Each of the phase I and II enzymes involved in estrogen metabolism possesses genetic variants that (*a*) are associated with altered enzyme function and (*b*) occur in a sizable portion of the population.^38^,^44^ We and others have determined the enzymatic rate constants (*k_cat_* and *K_m_*) of the common *CYP1A1*, *CYP1B1*, and *COMT* variants and compared their activitytotherespectivewild-typeenzymes.^26^,^29^,^32^,^45^,^46^ These studies were limited to individual enzyme reactions and did not take the entire estrogen metabolism pathway into account. To obtain a more realistic and inclusive view of estrogen metabolism in the female population, we utilized the model to simulate how variations in the kinetic parameters resulting from polymorphisms of the enzymes impact the metabolite concentrations. We examined 4 *CYP1A1*, 16 *CYP1B1*, and 2 *COMT* alleles. Thus, our simulations are based on the examination of 4·16·2 = 128 genetic combinations to demonstrate the utility of the model. Although each of the metabolites can be modeled, we concentrated our analysis on the catechols and quinones because of their documented carcinogenic activity.^15^,^22^

Since women may differ in their combination of enzyme variants, they will have different rate constants, resulting in differences of 4-*OHE*_2_ and *E*_2_-3,4-*Q* production. As shown in Figure 3 modeling of the 128 haplotype combinations produced a spectrum of catechol and quinone concentrations over time, as expressed by a range of *AUC* values. The simulations identified the haplotype combinations producing the highest and lowest *AUC*s. For example, the maximum *AUC*s for 4-*OHE*_2_ and *E*_2_-3,4-*Q* were produced by the haplotype *CYP1A1*_461_*_Asn_*_-462_*_Ile_* *CYP1B1*_48_*_Arg_*_-119_*_Ser_*_-432_*_Val_*_-453_*_Asn-_COMT*_108_*_Met_*, which were 2.6- and 4.6-fold higher, respectively, than the minimum *AUC*s produced by haplotype *CYP1A1*_461_*_Thr_*_-462_*_Val_* *CYP1B1*_48_*_Gly_*_-119_*_Ala_*_-432_*_Val_*_-453_*_Ser_COMT*_108_*_Val_*. While 2.6 to 4.6-fold differences may not appear large, it is important to consider that they impact on lifetime exposure, which is consistent with the hormonal risk model presented by Pike.^2^

**Figure 4:**
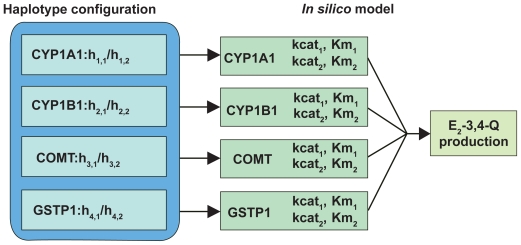
*Utilization of in silico model to derive E*_2_-3,4-*Q production*. Each of the four genes is genotyped for all subjects and the SNP genotype data used to derive the haploype configuration for each subject. The model then calculates the *E*_2_*-*3,4*-Q* production for each haplotype configuration as well as the weighted average of all *E*_2_-3,4-*Q* production values, using the probabilities of haplotype configurations as weights.

If a subject’s haplotypes can be resolved for all genes (i.e. she has at most one heterozygous SNP for each gene), then the *in silico* model can be used directly to derive the *E*_2_-3,4-*Q* production, as depicted in Figure 4. When a subject’s haplotype configurations are uncertain for some genes because of the presence of two or more heterozygous SNPs (e.g. *CYP1B1*), we first calculate the distribution of all haplotype configurations using PHASE^47^ (stephenslab.uchicago.edu/software.html). Then we derive the *E*_2_-3,4-*Q* production value for each haplotype configuration, and calculate the weighted average of all *E_2_*-3,4-*Q* production values, using the probabilities of haplotype configurations as weights. It can be shown that this weighted average is the expected *E*_2_-3,4-*Q* production given the genotypes. This way, we incorporate information from all genotyped SNPs and each haplotype configuration is apportioned appropriately. Application of the model to a breast cancer case-control population (438 pre- and postmenopausal women with 221 invasive breast cancer cases and 217 controls) defined the estrogen quinone *E*_2_-3,4-*Q* as a potential breast cancer risk factor. This exploratory analysis identified a subset of women at increased breast cancer risk based on their enzyme haplotype and consequent *E*_2_-3,4-*Q* production.^42^ Based on the *E*_2_-3,4-*Q AUC* values, cases predominated in the top tier of the population. For example, among the 10 women with the highest *E*_2_-3,4-*Q* values in the entire study population, there were nine cases and one control (p-value = 0.01). These results suggest for the first time the possibility that breast cancer risk prediction may be enhanced by incorporation of inherited differences in estrogen metabolism.

Obviously, the model requires testing and as a first step we have examined the contribution of the estrogen concentration on *E*_2_-3,4-*Q* production and the associated breast cancer risk. Numerous epidemiological studies have implicated estrogens in the development of breast cancer.^3^ For example, a pooled analysis of nine prospective studies of serum estrogen levels and breast cancer in 2428 postmenopausal women revealed a strong association of serum *E*_2_ concentrations with breast cancer risk.^4^ The relative risk of breast cancer for women whose free *E*_2_ levels were in the top quintile was 2.58 compared with 1.00 for those women whose levels were in the bottom quintile. Since the nine studies employed different methods to measure, *E*_2_, there were considerable differences in the median *E*_2_ values reported. In spite of this variability, the median serum *E*_2_ concentrations in seven of the nine studies were higher in the case patients than in the control subjects. To incorporate the different levels into our simulations, we introduced a ratio that is defined as *E*_2_ Ratio = *E*_2_0__^(^*^case^*^)^ /*E*_2_0__^(^*^control^*^)^. In Table 1, we summarized the median *E*_2_ values for the nine studies as well as the corresponding cases/controls *E*_2_ ratios, which ranged from 0.91 to 1.34. These ratios appear rather narrow and are of unknown biological significance. We used the model and our study population to determine whether such seemingly small differences in serum *E*_2_ concentrations between cases and controls could influence mammary estrogen metabolism sufficiently to cause significant differences in the production of the carcinogenic *E*_2_-3,4-*Q*. Since serum *E*_2_ was not measured in our study population, we used the initial level *E*_2_0__ for the cases and controls from the nine prospective studies to calculate the *E*_2_-3,4-*Q AUC* for the 294 postmenopausal women in our group. There were 144 women with breast cancer and 150 control subjects with average ages of 65.6 and 64.9 years and average body mass indices of 25.7 and 26.0 kg/m^2^, respectively. In our simulations we varied the *E*_2_ ratio between cases and controls from 0.91 to 1.34 and calculated the corresponding *E*_2_-3,4-*Q AUC* values. As the *E*_2_ Ratio = *E*_2_0__^(^*^case^*^)^ / *E*_2_0__^(^*^control^*^)^. varied, the fraction of cases in the top *E*_2_-3,4-*Q AUC* values of the women also changed. With an *E*_2_ Ratio of 0.908 (Rancho Bernardo), only 31 women in the top AUC cases and controls had breast cancer compared to 65 women (Washington Country) at 1.06 (p = 0.037) and 102 (SOF) at 1.34 (p < 0.00001). The results of these simulations demonstrate that relatively small changes in the concentration of the parent hormone *E*_2_ result in markedly increased production of the carcinogenic estrogen quinone metabolite, *E*_2_-3,4-*Q*, which, in turn, is reflected in a higher fraction of women with breast cancer in the top tier of our study population. Thus, testing of our model with estrogen concentrations reported in the literature confirms the striking influence of serum *E*_2_ concentrations on breast cancer risk. Importantly, the model offers a risk assessment of individual women by combining the hormone level with the genotype.

## Discussion

**Figure 5:**
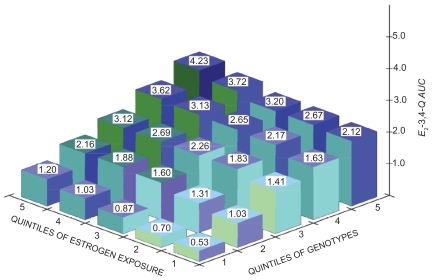
*Three-dimensional graph displaying estrogen metabolomic-genomic model of breast cancer risk.* The risk is represented by the amount of carcinogenic estrogen quinone*, E*_2_*-3,4-Q AUC*, which is produced by the metabolism of estrogen catalyzed by the enzymes *CYP1A1*, *CYP1B1*, and *COMT*. In theory, all enzyme genotype combinations could be plotted. However for clarity, we have plotted representative combinations from lowest to highest, separated into quintiles based on their respective *E*_2_*-3,4-Q* production: (1) *CYP1A1*_461_*_Thr_*_-462_*_Val_CYP1B1*_48_*_Gly_*_-119_*_Ala_*_-432_*_Val_*_-453_*_Ser_COMT*_108_*_Val_* (2) *CYP1A1*_461_*_Thr_*_-462_*_Val_CYP1B1*_48_*_Arg_*_-119_*_Ala_*_-432_*_Val_*_-453_*_Asn_COMT*_108_*_Met_*, (3) *CYP1A1*_461_*_Thr_*_-462_*_Ile_* *CYP1B1* _48_ *_Arg_*_-119_*_Ala_*_-432_*_Val_*_-453_*_Ser_COMT*_108_*_Val_*, (4) *CYP1A1*_461_*_Thr_*_-462_*_Ile_* *CYP1B1*_48_*_Arg_*_-119_*_Ala_*_-432_*_Val-_*_453_*_Ser_COMT*_108_*_Met_*, (5) *CYP1A1*_461_*_Asn_*_-462_*_Ile_CYP1B1*_48_*_Arg_*_-119_*_Ser_*_-432_*_Val_*_-453_*_Asn_COMT*_108_*_Met_*. Following the characterization of *GSTP1* variants, we will include *GSTP1* genotype data in the model. Cumulative estrogen exposure is displayed in quintiles. Actual *E*_2_ values, measured in pmol/L, could be plotted, in combination with semi-quantitative estimates of each woman’s overall exposure to estrogen. The latter is derived by taking into account her total years of ovulation as a function of current age, age at menarche, age at menopause, numbers of full-term pregnancies and lactation experience for each, and the dosage and duration of the use of exogenous estrogens. (The authors acknowledge work of Eric Parl in the design and preparation of this figure).

A strength of the *in silico* model is that it can incorporate each woman’s actual lifetime endogenous and exogenous estrogen exposures, in addition to her genotype, when predicting cumulative *E*_2_-3,4-*Q* exposure. This is schematically shown in Figure 5, which displays the interaction of estrogens, enzyme genotypes, and resulting *E*_2_-3,4-*Q* production as a three-dimensional graph. The graph is built on the two-dimensional Figure 3, in which we used a fixed *E*_2_ level to model the *E*_2_-3,4-*Q AUC* for wild-type and variant enzyme genotypes and displayed only the lowest, highest, and wild-type *E*_2_-3,4-*Q AUC*s. In the three-dimensional graph, we plot the available genotypes, from lowest to highest, separated into quintiles based on their respective *E*_2_-3,4-*Q* production. A new component in the three-dimensional graph is the variation in *E*_2_ concentration. As illustrated in the overall pathway in Figure 1 and in experimental studies, the input concentration of the parent hormone *E*_2_ determines the output concentration of the oxidative metabolites, such as 4-*OHE*_2_ and *E*_2_-3,4-*Q*.^26^,^29^,^32^,^45^,^48^ Thus, in the graph we display estrogen exposure in quintiles. Estrogen exposure can be represented by actual *E*_2_ values, measured in pmol/L, in combination with semiquantitative estimates each woman’s overall exposure to estrogen. The latter is derived by taking into account her total years of ovulation as a function of current age, age at menarche, age at menopause, numbers of full-term pregnancies and lactation experience for each, and the dosage and duration of the use of exogenous estrogens. With regard to exogenous estrogens, all estrogens including equine estrogens used in hormone replacement therapy are metabolized via the same *CYP*-mediated oxidative pathway to generate catechols and quinones, which, in turn, cause DNA damage. For example, cell culture experiments showed that *4-OH*-*equilenin* via its quinone induced DNA damage in breast cancer cell lines and cellular transformation in vitro.^49^,^50^ Thus, as far as the model is concerned, exogenous and endogenous estrogens can be combined although their precise contribution to estrogen exposure and the production of carcinogenic metabolites is presently unknown.

In designing Figure 5, we assumed that the difference in estrogen exposure between individual women is no more than twofold, with the quintiles 1.0, 1.25, 1.5, 1.75, 2.0. We chose this two-fold difference based on the range of median serum *E*_2_ values seen in post-menopausal women^4^ and the variation in mammary tissue *E*_2_ concentrations.^5^ This range is conservative since up to fivefold differences have been reported.^52^ Regardless of the scale used for the estrogen exposure axis, the production of carcinogenic *E*_2_-3,4-*Q* would be expected to be greater in women with more endogenous (more ovulatory cycles) or exogenous (hormone replacement therapy, oral contraceptives) estrogen exposure. It is evident from Figure 5 that our *in silico* model extends existing models by combining the traditional “phenotypic” measures of estrogen exposure with genotypic data. It is also evident from the 3D graph that the combined phenotypic and genotypic data appear to have not just an additive, but also a multiplicative effect on *E*_2_-3,4-*Q* production.

Current models of breast cancer risk prediction are mainly based on cumulative estrogen exposure but do not reflect mammary estrogen metabolism;^16^ (www.cancer.gov/bcrisktool). Moreover, they do not address genetic variability between women in exposure to estrogen metabolites. Our model addresses the unique genetic trait of each woman and combines the genetic information with the metabolomic information in order to predict individual-level mammary estrogen metabolism. Some genetic traits are currently available in the patient care setting, such as BRCA and CYP2D6 testing. The availability of rapid genetic testing for BRCA1 and BRCA2 mutations has made it possible to follow unaffected carriers in greater numbers and to search for inherited mutations in women with a severe family history of breast cancer. The potential effect of CYP2D6 genetic variants on clinical response to tamoxifen treatment in breast cancer patients has gained much interest.^53^ The Food and Drug Administration recommended an update in the tamoxifen package insert in 2006 to reflect the increased risk of breast cancer recurrence in postmenopausal estrogen receptor-positive patients, who are CYP2D6 poor metabolizers. Thus, the CYP2D6 genotype has the potential to become a useful predictive marker for tamoxifen response. Certain characteristics are beneficial for a marker to become successful clinically.^54^ Testing of this marker should be cost-effective as well as easy to apply in daily practice, both of which are increasingly realized for DNA analysis. Thus, analysis of multiple genes encoding the enzymes in the estrogen metabolism pathway can readily be achieved. Estrogen is a universal breast cancer risk factor; by helping to define high-risk subgroups, the proposed model should advance the overall goal of reducing breast cancer mortality through improved screening and the early detection and treatment of disease. Rates of obesity, an important source of estrogen after menopause are on the rise in most of the world, underscoring the importance of establishing the impact of estrogen metabolites on breast cancer risk before and after menopause. Women who carry a germline mutation of BRCA1 frequently develop breast cancer at an early age. However, in any given kindred the age of onset can vary substantially and an important unresolved question is the extent to which other risk factors modify the cancer risk in carriers. Estrogen exposure appears to play an important role since prophylactic oophorectomy is associated with a significant reduction in the risk of breast cancer.^55^ A practical clinical application of the model in the premenopausal age group would be the differentiation of BRCA1 carriers into low- and high-risk based on their genetic profile of estrogen metabolism. Another clinical application of the model would be in the postmenopausal age group with the distinction of low- and high-risk women. The former could benefit from hormone replacement therapy whereas the latter should avoid such treatment.

In summary, our *in silico* model integrates pathway-specific genetic testing with diverse types of data and for the first time offers the opportunity to combine exposure, metabolic, and genetic data in assessing estrogens in relation to breast cancer risk. In order to achieve such comprehensive risk assessment, the model will require extensive validation.

## Figures and Tables

**Table 1:** Correlation of postmenopausal serum estradiol concentration by study and case-control status with breast cancer risk. Controls (m in number) and cases (n in number) are individuals with the top AUC values (m + n) in the simulation model. The case-control data is from^42^ and the *E*_2_ Ratio is taken from a reanalysis of nine pooled prospective studies.^4^ The ratio of all centers is the average ratio of the nine centers.

Study, country	Estradiol pmol/L			m	n	n/(m + n)	
	
	Cases	Controls	Ratio	Cases	Controls	% Cases	p-value
Columbia, MO United States	55.1	51.4	1.07	63	42	60.0	0.034
Guernsey, United Kingdom	45.5	35.0	1.30	100	4	96.2	<0.001
Nurses’ Health Study, United States	29.4	25.7	1.14	77	31	71.3	<0.001
NYU WHS, United States	134	101	1.33	102	4	96.2	<0.001
ORDET, Italy	21.9	21.7	1.01	59	55	51.7	<0.1
Rancho Bernardo, United States	36.7	40.4	0.908	31	73	29.8	<0.1
RERF, Japan	63.1	64.5	0.978	39	69	36.1	<0.1
SOF, United States	29.4	22.0	1.34	102	4	96.2	<0.001
Washington Country, United States	62.4	58.7	1.06	65	44	59.6	0.037
Average of all centers			1.126	78	35	69.0	<0.001

## Appendix

**Table:** Nomenclature

Symbol	Quantity
*E*_2_	17β-estradiol
*OHE*^2^_2_	2*-OHE*_2_*-*catechol estrogen
*OHE*_2_^4^	4*-OHE*_2_*-*catechol estrogen
*MeOHE*^2^_2_	2*-MeOE*_2_-methoxyestrogen
*MeOHE*_2_^23^	2*-OH-3-MeOE*_2_-methoxyestrogen
*MeOHE*_2_^4^	4*-MeOE*_2_-methoxyestrogen
*OHE*_2_^21^*SG*	2-OHE_2_-1-SG-GSH-conjugate
*OHE*_2_^24^*SG*	2-OHE_2_-4-SG-GSH-conjugate
*OHE*_2_^42^*SG*	4-OHE_2_-2-SG-GSH-conjugate
*EQ*_2_^23^	*E*_2_*-*2,3*-Q*-quinone
*EQ*_2_^34^	*E*_2_*-*3,4*-Q*-quinone
*t*	time
$\int_{0}^{T}{EQ}_{2}^{34}dt$	Area under the Curve (*AUC*)
*E**_CYP_*_1_*_A_*_1_, *E**_CYP_*_1_*_B_*_1_, *E**_COMT_*, *E**_GSTP_*_1_	Enzyme Concentrations
*k**_cat_*_*_i_*_, *i* = 1, . . . , 14	Rate Constants
*K**_m_*_*_i_*_*, i,*1, 2, . . . ,14	Rate Constants
*k*_1_*and k*_2_	Rate Constants for decay of Quinones
*α* and *β*	Hill exponents
*V*_max*_Q_*_1__, *V*_max*_Q_*_2__, *K**_m_*_*_Q_*_1__, *K**_m_*_*_Q_*_2__	Rate Constants for Quinone Formation
